# Colonic Microbiota Profile Characterization of the Responsiveness to Dietary Fibre Treatment in Hypercholesterolemia

**DOI:** 10.3390/nu14030525

**Published:** 2022-01-25

**Authors:** Ana Belen Granado-Serrano, Meritxell Martín-Garí, Virginia Sánchez, Marissa Riart Solans, Antonia Lafarga Giribets, Rebeca Berdún, Ester Vilaprinyó, Manuel Portero-Otín, José C. E. Serrano

**Affiliations:** 1 NUTREN-Nutrigenomics, Department of Experimental Medicine, University of Lleida, 25198 Lleida, Spain; anabgs@gmail.com (A.B.G.-S.); meritxell.martin@udl.cat (M.M.-G.); rebecaberdun@gmail.com (R.B.); manuel.portero@udl.cat (M.P.-O.); 2 Institut Català de la Salut, 08028 Barcelona, Spain; virginia.sanchez@gmail.com (V.S.); mriart.pirineu.ics@gencat.cat (M.R.S.); alafarga@gmail.com (A.L.G.); 3 Department of Basic Medical Sciences, University of Lleida, 25198 Lleida, Spain; ester.vilaprinyo@udl.cat

**Keywords:** dietary fibre, cholesterol, blood lipid profile, colonic microbiota

## Abstract

This study aimed to determine how the microbiota profile might be predisposed to a better response in blood lipid profiles due to dietary fibre supplementation. A three-arm intervention study that included three different fibre types (mainly insoluble, soluble, and antioxidant fibre) supplemented (19.2 g/day) during 2 months in individuals with hypercholesterolemia was developed. Changes in faecal microbiota and blood lipid profile after fibre supplementation were determined. In all volunteers, regardless of fibre type, an increase in the abundance of *Bifidobacterium* was observed, and similarly, an inverse relationship between faecal propionic acid and blood LDL-cholesterol, LDL particle size, and LDL/HDL particle ratio (*p*-values 0.0067, 0.0002, and 0.0067, respectively) was observed. However, not all volunteers presented an improvement in lipid profile. The non-responders to fibre treatment showed a decrease in microbiota diversity (Shannon and Simpson diversity index *p*-values of 0.0110 and 0.0255, respectively) after the intervention; where the reduction in short-chain fatty acids (SCFAs) producing bacterial genera such as *Clostridium XIVa* and *Ruminococcus* after dietary fibre treatment was the main difference. It was concluded that the non-responsiveness to dietary fibre treatment might be mediated by the lack of ability to maintain a stable SCFA producing bacteria diversity and composition after extra fibre intake.

## 1. Introduction

In recent years, several intervention trials focusing on the cholesterol-lowering effects of fibre-rich diets have been developed [1]. In particular, it has been observed that people following a diet with a low fibre intake (around 14 g/day) have a 23% higher cardiovascular mortality risk compared with a higher fibre intake (around 30 g/day) [2]. Nevertheless, the data on the effects of fibre supplementation on plasma lipids are still limited and controversial; some of them show lipid profile improvements that did not reach statistical significance [3,4].

Similarly, gut microbiota has emerged as an important player in host lipid homeostasis [5,6]. For instance, the microbial diversity and several taxa of the gut microbiota, like *Fusobacteria*, and a low abundance of *Oscillospira* have been associated with hypertriglyceridemia [7], whereas faecal isobutyric acid content correlated positively with *Odoribacter* and an unfavourable lipid profile [6]. In this sense, differences in microbiota profile and dietary fibre intake between subjects may predispose to variability in the lipid metabolism response to dietary fibre supplementation. Therefore, the modulation of the gut microbiota via dietary approaches could be a promising option for improving dyslipidemia.

Several studies have determined the dietary fibre modulation capacity of gut microbiota. Most of them focus on the analysis of changes in the profile of the microbiota towards an increase in bacteria considered beneficial, as opposed to bacterial genera considered harmful [8]. It is concluded that the main effect of extra fibre consumption is an increase in species of the genus *Bifidobacterium* [9,10,11]. However, no modifications are observed in other bacterial genera of interest, such as butyrate-producing bacteria, for instance, *Ruminococcus*, which could limit changes in the production of SCFAs [12,13]. Some authors suggest that despite the increased availability of fermentable carbohydrates of diverse physicochemical structures through a high-fibre diet, only a small number of bacteria with the genetic capacity to produce short-chain fatty acids (SCFAs) was able to take advantage and become the dominant positive responders [14]. It is for this reason that some researchers suggest the importance of the initial microbiota profile in determining individual responsiveness to fibre supplementation.

Modulating gut microbiota by dietary interventions becomes a potentially promising strategy to demonstrate this chain of causation of the role of gut microbiota in dyslipidemia. However, it remains unknown whether the baseline profile of gut microbiota could modify the response of dietary fibre intake in disease treatment and prevention. In this sense, to improve the understanding of the efficacy of dietary fibre interventions, it is critical to understand how members of the gut ecosystem respond when exposed to increased dietary fibre intake as a new environmental resource.

This study aimed to determine how the initial microbiota profile might predispose to a better response to dietary fibre supplementation and its relationship with the observed changes in blood lipid profile in a hypercholesterolemic population. For that purpose, a three-arm parallel intervention study was developed where volunteers in each arm were supplemented with 19.2 g/day and one of three different types of fibre (rich in insoluble, soluble, and antioxidant fibres) for 2 months. As a primary outcome, the baseline and changes in microbiota and blood lipid profile after fibre treatment were determined. With this information, the understanding of the relationship between the microbiota and dietary fibre was addressed in two ways: (1) to determine if the initial differences in the microbiota profile are predisposed to changes in the blood lipid profile after dietary fibre treatment, and (2) to determine if the people who responded better to fibre treatment had a differential microbiota profile from those who did not observe any response.

## 2. Materials and Methods

### 2.1. Study Design

For a randomized parallel trial where volunteers were randomly allocated to three different arms, (1) insoluble, (2) soluble, and (3) antioxidant fibre was designed. The intervention consisted of daily intake for two months of six fibre-rich cookies (93 g/day) that provided a total intake of 19.2 g/day of dietary fibre. Insoluble fibre cookies (wheat bran) consisted of fibre-rich cookies manufactured by the Sanavi S.A. (Láchar, Granada, Spain) product catalogue (20.18217/GR), while in Soluble and Antioxidant fibres, 5 g of Psyllium plantago (Association Nature and Partage, Gironde-sur-Dropt, France) or onion-based antioxidant fibre (BIOMED, Lleida, Spain; European Patent number EP2936999A1 and USA Patent Number US9.700.576 B2), respectively, were substituted by 5 g of wheat bran of the original commercial formulation. The nutritional composition of each preparation was the same for the three types of cookies, as described in Table S1 in the Supplementary Materials.

Participants were instructed to maintain their lifestyle habits and diet and to divide the intake of the cookies three times during the day. All participants and staff involved in the recruitment and analyses of outcome variables were blinded to the intervention allocation. The protocol was registered at ClinicalTrials.gov (NCT0454563). The study was conducted according to the ethical guidelines of the Helsinki Declaration, and the study protocol was approved by the Clinical Research Ethics Committee of the Institut Catalá de Salut from Hospital Universitari Arnau de Vilanova in Lleida, Spain (CEIC-1534. 21/12/2015).

After the intervention, changes in the lipid profile were analyzed and the volunteers were divided into two groups: (1) responders to fibre treatment, and (2) non-responders to fibre treatment, where the type of fibre supplement did not matter in both cases. The division of the volunteers into responders and non-responders was based on the expected change in cholesterol levels from treatment with dietary fibre according to meta-analyses published in the Cochrane database. It is suggested that dietary fibre, regardless of the type of fibre, induces a significant reduction in cholesterol levels between 2.3 to 15.4 mg/dL [3]. Accordingly, volunteers who experienced a decrease in total cholesterol ≥ 2.3 mg/dL were considered as responders to dietary fibre treatment. The remaining volunteers who experienced slight modifications or increased levels of total cholesterol after treatment were identified as non-responders.

### 2.2. Sample Size Calculation

The sample size was calculated based on the study published by Pereira et al. [15], in which a standard deviation of 4.8 mg/dL was estimated in cholesterol levels within persons, and in subjects with hypercholesterolemia. The expected outcome after fibre supplementation was a reduction in 8.9 mg/dL in total blood cholesterol levels, following the results of the Cochrane systematic review [3]. The number of volunteers needed in each intervention group for a two-sided test, a 0.05 Type I error rate, and an estimated power of 0.99 was 11. Taking into consideration a possible dropout of 30%, the recruitment goal was 15 volunteers per study intervention group.

### 2.3. Subjects

**Figure 1:**
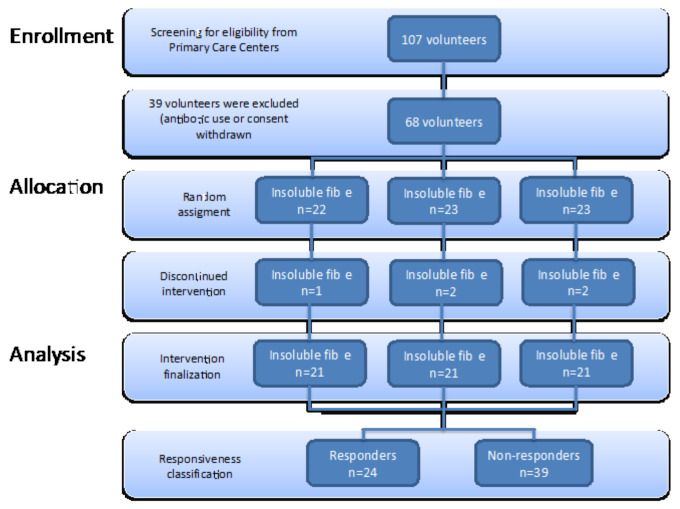
Flow chart of study design and volunteer recruitment.

The inclusion criteria included volunteers from both sexes and ages between 18 to 65 years old, diagnosed with hypercholesterolemia (more than three blood test analyses with high levels of total cholesterol (>200 mg/dL)). Volunteers were recruited at Primary Care Centers in Catalonia, with the collaboration of the Center’s Family Physicians. The exclusion criteria included: diagnosed metabolic pathologies, such as type 1 or type 2 diabetes, lipid-lowering drug prescription, and probiotic supplements and/or antibiotics in the last two months. All volunteers were adequately informed before giving their consent. The flow chart of the study design and volunteer recruitment is described in Figure 1. In total, 63 volunteers aged between 37–68 years from Lleida (Spain), whose demographic data are provided in Supplementary Table S2, completed the study.

### 2.4. Blood Biochemistry

Venous blood samples in fasting conditions (12 h) were collected in serum separator tubes (BD, Ref. 367953). Serum was collected immediately after clotting by centrifugation for 15 min at 4 °C and 1500× *g*. Then, the serum was stored and kept at −80 °C until further biochemical analysis. Total cholesterol (TC) and Triacylglycerides (TG) levels, as well as their content in lipoproteins (VLDL: very low-density lipoprotein, IDL: intermediate-density lipoprotein, HDL: high-density lipoprotein, LDL: low-density lipoprotein), the average size of each particle, and particle number were measured through the Liposcale test by the Research Unit on Lipids and Atherosclerosis of Sant Joan University Hospital and Universitat Rovira i Virgili, at Reus (Spain), following the procedures described by Mallol and colleagues [16].

### 2.5. Microbial DNA Purification, 16S Amplicon Preparation, Sequencing, and Processing

A single stool sample produced at any time of the day before blood extraction, with no specific dietary restrictions, was requested. Samples were collected in sterile plastic containers (Deltalab, Barcelona, Spain, Ref. 4097226) and kept at −20 °C until their delivery. Once in the laboratory, samples were divided into aliquots of approximately 200 ± 20 mg and frozen at −80 °C for further analysis. Microbial DNA was extracted after a previous bead-beating step (1 cycle of 40 s at 4 °C) in Lysing Matrix E tubes for FastPrep 24 (MP Biomedicals. 11452420) with the QIAamp DNA Stool Mini Kit (Qiagen, Hilden, Germany, Ref. 51504) following the manufacturer’s instructions. DNA quality and yield were determined via 1% (*w*/*w*) agarose gel electrophoresis and NanoDrop 2000 UV spectrophotometer (Thermo Fisher Scientific, Waltham, MA, USA). Bacterial populations were determined using next-generation high-throughput sequencing of variable regions of the 16S rDNA bacterial gene using 16S universal primers targeting the V3–V4 region with 16S rRNA gene libraries generated by Vaiomer (Tolouse, France). The joint pair length was set to encompass 476 base pair amplicons thanks to the 2 × 300 paired-end MiSeq kit V3. For each sample, a sequencing library was generated by the addition of sequencing adapters. The detection was performed using MiSeq Illumina technology. FROGS v1.3.0 guidelines were used for the analysis of targeted metagenomics sequences, and clustered into OTUs with the Swarm algorithm before taxonomic assignment [17]. OTUs identified as chimera (relative abundance lower than 0.005% in the whole dataset identified by vsearch v1.9.5), or with a strong similarity (coverage and identity ≥ 80%) with the phiX (Illumina, San Diego, CA, USA) were removed. Clustering was produced in two passes of the swarm algorithm v2.1.6. The first pass was a clustering with an aggregation distance equal to 1, and the second one, equal to 3. The taxonomic assignment was produced by Blast +v2.2.30+ with the databank RDP v11.4. In total, 997 OTUs were available for further analysis after filtering.

### 2.6. Bacterial Analysis from 16S rRNA Data

#### 2.6.1. Species Diversity

Species diversity was assessed through: (1) the number of different taxa observed (OTUs observed), which refers to the actual richness observed. (2) Chao1 index, which estimates the richness of a community based upon the number of rare species that may have been missed due to under-sampling. (3) Shannon’s index, which represents the average certainty to predict the identity of unknown individuals, and (4) Simpson’s index, that is based upon the probability that two randomly selected individuals will belong to the same species. Species richness and diversity indexes were calculated using the vegan Community Ecology Package in R programming [18].

#### 2.6.2. Taxonomic Composition of Fecal Microbiota Profile

OTU data from each group were compared by the linear discriminant analysis effect size (LEfSe) algorithm [19] in the Galaxy/Metabiome portal. A *p*-value < 0.05 was considered as significant. Results were plotted on a Cladogram. Differences in the relative abundance of each bacterial taxon (phylum, class, order, family, genus, and species) were also assessed by the function runWilcox in the R environment to obtain the *p*-value adjustment of each comparison for multiple testing (*q*-value).

### 2.7. Prediction of Functional Metagenome (PICRUSt Analysis)

The functional metagenome (microbiome) of the fecal bacterial community in each studied population was predicted based on the 16S rRNA sequence data using the PICRUSt approach (phylogenetic investigation of communities by reconstruction of unobserved states) from the Galaxy Hutlab server and the Kyoto Encyclopedia of Genes and Genomes (KEGG) pathways as a reference database. A total of 269 gene family pathways were identified.

### 2.8. Faecal Short-Chain Fatty Acids Profile

The short-chain fatty acids (SCFA) profile present in the feces was determined by high-performance liquid chromatography (HPLC-VWD) [20], detected as acid hydrazides at 400 nm. A frozen sample of approximately 200 mg was placed in a weighed glass centrifuge tube, and 5.0 mL of 70% ethanol was immediately added. The tube was weighed to determine the fecal weight. Further samples were mixed and centrifuged at 20 °C, 2500 rpm for 10 min, and the supernatant was collected. An aliquot of 300 μL of each sample was added with 50 µL of 2-ethylbutyric acid as the internal standard, and then derivatized with 300 µL of pyridine. 1-EDC-HCl and 2-NPH-HCl were reaction-assistive agents, reacted at 60 °C for 20 min, and then the reaction was stopped by the addition of 200 µL potassium hydroxide and incubated at 60 °C for 20 min. After cooling, the mixture was shaken with 3 mL of phosphoric acid aqueous solution and 4 mL of ether for 3 min, and then centrifuged. The obtained ether layer was shaken with 4 mL of water for 3 min and then centrifuged. The obtained ether layer was evaporated using nitrogen gas. Finally, the residue which contained fatty acid hydrazide was dissolved with 100 µL of methanol, and 30 µL was subjected to HPLC. All standards and reagents were purchased from Sigma-Aldrich. The analysis was performed using an Agilent Technologies Series 200 HPLC with an YMC-Pack FA 250 × 6 mm ID column. Column temperature was set at 50 °C with a flow rate of 1.1 mL/min, and fatty acid hydrazides were detected at a wavelength of 400 nm.

### 2.9. Dietary and Nutritional Parameters

At each visit, 24 h total dietary recall obtained with the collaboration of trained dieticians was registered. The daily amounts of nutrients ingested by each volunteer and the total energy intake were estimated through DIAL software (Alceingeniería S.A., Madrid, Spain). Additionally, blood pressure was taken using an automatic blood pressure cuff. Weight and height were measured using a weight scale with an incorporated steel ruler for height determination with an accuracy of 0.1 kg and 0.1 cm, respectively. The skinfolds thickness and estimation of total body fat were measured with a calliper, with an accuracy of 0.1 mm. The remaining anthropometric measurements were performed using a flexible measuring tape with an accuracy of 0.1 cm.

### 2.10. Statistical Analysis

Data are presented as mean ± standard deviation, range (min–max), and 95% confidence interval of the mean (95% CI min–max). Two-tailed Student’s *t*-tests were performed for the comparisons between the two groups. Statistical comparisons of bacterial taxons were performed by LEfSe analysis in the Galaxy/Metabiome portal. Relative abundances were estimated with the function runWilcox from the EMA package in an R environment, which adjusts the *p*-value for multiple testing. Diversity indexes and metadata (lipid biomarkers, anthropometric data, dietary parameters, and SCFAs) were compared by the unpaired *t*-test or Wilcoxon Mann-Whitney U test in an R environment, considering a *p*-value < 0.05 as significant. P-adjustment after false discovery rate correction from multiple comparisons was performed with the runTtest or runWilcox function when more than 10 variables of the same family data set were evaluated, and *q*-values were added. Correlation analysis was calculated by Pearson’s correlation method in an R statistical framework using the cor.test function from the stats package corrplot. *p*-values were adjusted for multiple comparisons; a *q*-value considered as significant is indicated in the legend of each figure. Results were plotted in an R environment using the ellipse package.

## 3. Results

### 3.1. Study Population Characteristics at Baseline

The mean cholesterol levels observed at the beginning of the study was 208 mg/dL (95% CI between 202–215 mg/dL), with values in the upper adequate limit of LDL-cholesterol and normal values of TG. Lipoprotein particle size analysis was characterized by lower levels of small LDL and large HDL particles, and a low mean HDL particle size (HDL-z), which is associated with atherosclerosis [16,21]. Regarding dietary factors that may affect the lipid profile, at the beginning of the study, a higher intake of dietary lipids was observed, mono-unsaturated fatty acids being the main component of dietary lipids, while the fibre intake was below the intake recommendation (above 25 g/day).

Microbiota profile was characterized by a *Firmicutes*/*Bacteroidetes* ratio of 1.026 (95% CI of the mean 0.913–1.139) with normal distribution between volunteers (D’Agostino-Pearson omnibus normality test K2 = 3.809, *p* = 0.1489). At genus-level, data from 16S rRNA gene-sequencing gave three enterotypes characterized by a predominance of (1) *Bacteroides*; (2) *Prevotella*; and (3) non-specific predominance (Supplementary Figure S1A–D). *Bacteroides* were positively correlated with *Flavonifactor*, *Clostridium XIVa*, and *Roseburia*, and negatively with *Prevotella*, *Alloprevotella*, *Oscillibacter*, and *Megasphaera*. Similarly, *Prevotella* positively correlated with *Phenylbacterium*, *Megasphaera* and, *Enterobacter*, and negatively with *Bacteroides*, *Alistipes*, *Ruminococcus*, and *Clostridium XIVa* (Supplementary Figure S1E,F).

### 3.2. Global Effects of Dietary Fibre Supplementation in Lipid Profile

Volunteers that completed the study reported a mean consumption of fibre-rich cookies of 273 units (95% CI between 257–289) during the two months of intervention (a mean intake of 14.6 g of dietary fibre/day), which corresponds to 76% of the study’s adherence. Total dietary fibre intake (diet + fibre-rich cookies) during the intervention was around 34.1 g/day. There were no adverse reactions reported during the intervention.

The main effects of two-month dietary fibre supplementation of each dietary fibre group are described in Table S3 of the supplementary information. Regarding the type of fibre, wheat bran fibre and onion-based antioxidant fibre did not induce significant changes in any parameter of the lipid profile. Regarding the cookies with Psyllium plantago soluble fibre, an increase in TG levels in IDL and LDL lipoproteins and an increase in cholesterol levels in IDL lipoproteins were observed. Similarly, the increase in TG in LDL particles in the Psyllium plantago group was significantly different from the observed reduction in the onion-based antioxidant fibre group. Nevertheless, as a general result, no significant differences, or at least no improvement in blood lipid profile was found between groups and the treatment period.

The main effects of the two-month dietary fibre supplementation in all volunteers, independently of fibre type, are described in Table S4. Taken as a whole, fibre treatment did not induce changes in dietary and nutritional intake, nor in fecal SCFA composition. Notwithstanding, an increase in waist circumference and waist/hip ratio, as well as body fat percentage (mainly due to the increase in biceps and suprailiac skinfold thickness) was observed. As a favourable outcome due to fibre treatment, a reduction in blood pressure (Cohen’s d = 0.21) was observed. Regarding the blood lipid profile, unfavourable outcomes were observed, mainly due to the increase in small LDL and non-HDL particles, and lipoprotein particle ratios of total/HDL and LDL/HDL. Notwithstanding, the main concern of this study was the observation that fibre supplementation did not induce a favorable change in blood lipid profile.

**Figure 2:**
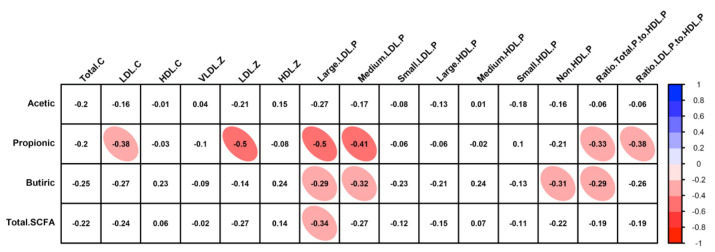
Pearson’s correlation analysis between changes in the blood lipid and SCFA profile. Correlations with *q*-values below 0.05 after adjustment for multiple analyses are highlighted with an ellipse. The color and slope of the ellipse indicate the magnitude of the correlation, with Pearson’s r value superimposed on the ellipse. The ellipses of negative correlations are shown in red. Correlations with *q*-values > 0.05 are in white.

To determine which of the volunteer’s characteristics may be predisposed to a favourable outcome in the lipid profile, a Pearson’s correlation analysis was performed between the observed changes in all studied parameters and the changes in lipid profile. The change in SCFAs in stool samples, mainly propionic and butyric acid, correlated with several parameters of blood lipid profile (Figure 2). Specifically, an inverse relationship was observed with the increase of propionic acid and the reduction in LDL-cholesterol, LDL particle size, and LDL/HDL particle ratio.

### 3.3. Responsiveness to Dietary Fibre Treatment Based on Initial Microbiota Profile

To determine whether a specific initial microbiota profile may be predisposed to a differential response to dietary fibre, the changes in blood lipid profiles and SCFAs were analyzed by the predominance of two main enterotypes, *Bacteroides* or *Prevotella*. Baseline and two-month treatment values are described in Table 1. The main basal difference observed between both microbiota’s enterotypes was the higher SCFA’s content in the *Prevotella* predominant group (*p* = 0.0008). Nevertheless, no differences in the percentage of acetic, propionic, and butyric acids were observed between enterotypes. This difference remained after dietary fibre treatment (*p* = 0.0015), where higher levels of total SCFAs were still observed in the *Prevotella* group, although fibre treatment induced a higher proportion of propionic acid (*p* = 0.0013). Regarding blood lipid profile, only the *Prevotella* enterotype presented significant changes in the blood lipid profile, mainly by the observed reduction in HDL cholesterol (*p* = 0.0012) and LDL-z particle size (*p* = 0.0070). Nevertheless, these differences were not observed between both enterotypes after dietary fibre treatment, suggesting that the initial predominant enterotype might not predispose to a differential response to dietary fibre treatment. 

### 3.4. Characteristics of Responders to Dietary Fibre Intake

Based on the last observation, it was suggested that the response to dietary fibre intervention might be modulated due to different characteristics or responses of the gut microbiota minority phyla. Therefore, volunteers were divided into two groups based on the response to dietary fibre treatment. Accordingly, volunteers who experienced a decrease in total cholesterol ≥ 2.3 mg/dL were considered as responders (*n* = 24) to dietary fibre treatment. The remaining volunteers who experienced slight modifications or increased levels of total cholesterol were identified as non-responders (*n* = 39). Basal characteristics and treatment effects of both groups are described in Supplementary Table S5.

**Figure 3:**
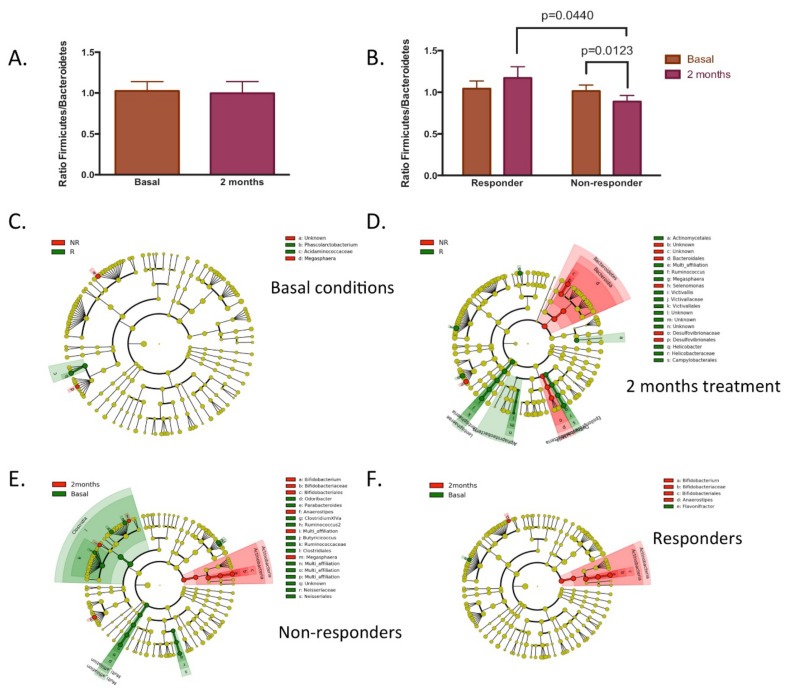
Bacterial taxonomic composition determined by 16S RNA analysis in fecal samples of responders and non-responders to dietary fibre treatment. (**A**) *Firmicutes*/*Bacteroidetes* ratio in all volunteers (*n* = 63) at the beginning and after 2 months of dietary fibre supplementation. (**B**) *Firmicutes*/*Bacteroidetes* ratio in responder and non-responder groups to dietary fibre treatment. (**C**–**F**) Cladogram plot of discriminant taxa between responders (R) and non-responders (NR) to dietary fibre treatment identified by LEfSe analysis (identified taxa presented a *p*-value of < 0.05). (**C**,**D**) Differences between responders and non-responders; (**E**,**F**) the changes observed within groups at the beginning and after two months of dietary fibre supplementation.

The main difference observed was a higher dietary fibre intake observed in the responder group. Dietary fibre treatment did not induce changes in the ratio of *Firmicutes*/*Bacteroidetes* (Figure 3); however, it was observed that the non-responder group presented a decrease in the ratio of *Firmicutes*/*Bacteroidetes* after two months of dietary fibre treatment, and was also lower compared to the responder group, in which no differences were observed. To this respect, few differences were observed in the taxonomic composition between both groups at the beginning of the study, with a higher ratio of main differences in the genera of *Phascolarctobacterium* and lower in the *Megasphaera* in the responder group (Figure 3C). After dietary fibre treatment, higher differences in fecal taxonomic composition were observed in both groups (Figure 3D), mainly by the observed increase in the genera *Ruminococcus*, *Helicobacter*, *Victivallis*, and *Megasphera*, and the decrease in *Selenomonas* for the responder group. The analysis of the taxonomic change due to fibre treatment in each group is shown in Figure 3E,F. Both responders and non-responder groups presented an increase in the ratio in the phylum of *Actinobacteria*, mainly in the *Bifidobacterium* genus, which could be considered as the main dietary fibre treatment effect. On the contrary, the non-responder group presented reductions in the abundance of *Anaerostipes, Clostridium XVIa, Ruminococcus2*, *Butyricoccus*, *Parabacteroides*, and *Odoribacter*; while the responder group presented a reduction in *Flavonifractor*.

Differences in Shannon and Simpson species diversity indexes were observed after dietary fibre treatment (Table 2). Nevertheless, the non-responder group was the only one that presented a significant reduction in the indexes described previously. In this sense, it was observed that fibre treatment did not induce major changes in the fecal taxonomic composition of the responder’s group, while the reduction in species diversity in the non-responder group could probably be attributed to the observed reduction in the *Clostridia* class, as described previously.

**Figure 4:**
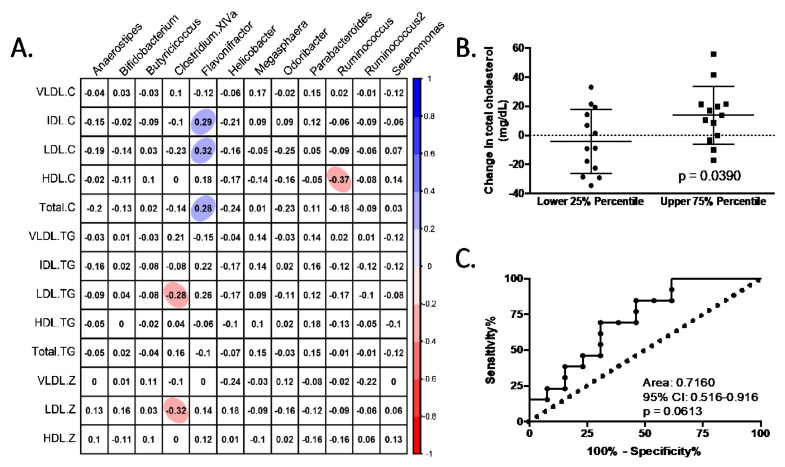
Differential genus between responders and non-responders to dietary fibre treatment and its relationship with changes in blood lipid profile. (**A**) Pearson correlation analysis between the changes in blood lipid and differential stool bacterial genus. Correlations with *q*-values below 0.05 after adjustment for multiple analyses are highlighted with an ellipse. The color and slope of the ellipse indicate the magnitude of the correlation, with Pearson’s r value superimposed on the ellipse. The ellipses of positive correlations are shown in blue, and the negative correlations are in red. Correlations with *q*-values > 0.05 are in white. (**B**,**C**) Receiver operating characteristics (ROC) analysis of the prediction capacity of the changes in *Flavonifractor* abundance due to dietary fibre treatment and the change in blood total cholesterol level. The analysis was performed whilst considerating volunteers that presented changes in feces *Flavonifractor* abundance below and above the 25th and 75th percentile. *P*-value in (**B**) was obtained by unpaired *t*-test analysis.

The next step was to determine whether the differences in bacterial genera before and after fibre treatment and between both groups (responders vs. non-responders) were correlated with the observed changes in the lipid profile (Figure 4). Of the bacterial genera that showed significant differences, *Flavonifractor* was positively correlated with total cholesterol and cholesterol in the IDL and LDL particles, while *Ruminococcus* negatively correlated with cholesterol levels in HDL particles. On the other hand, *Clostridium XIVa* was negatively correlated with the levels of TG in LDL particles and the size of LDL particles (LDL-z) (Figure 4A). To determine the degree of prediction of the reduction in total blood cholesterol levels based on the changes of Flavonifractor in feces, a receiver operating characteristic (ROC) analysis was performed (Figure 4B,C). In this sense, a change in *Flavonifractor* may have an accuracy of 0.7160 (ROC area) for the prediction of changes in blood cholesterol levels.

**Figure 5:**
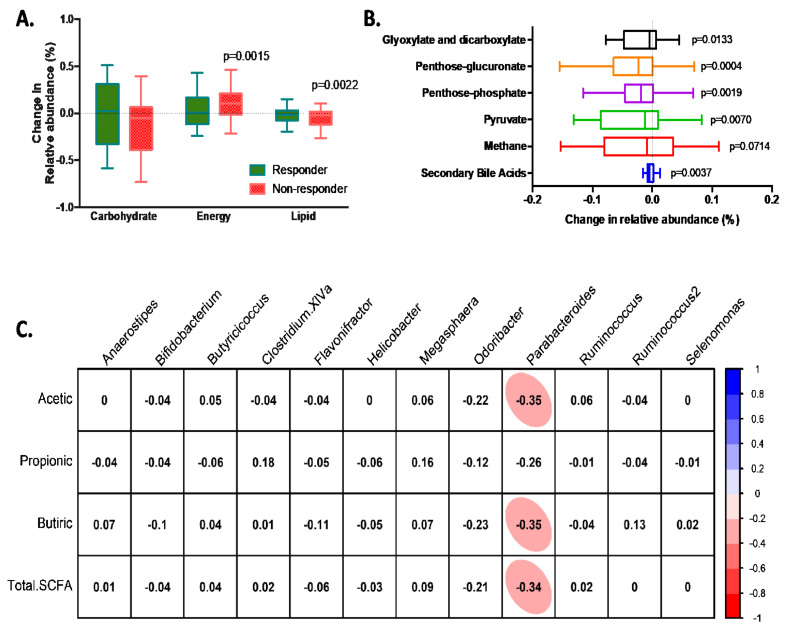
PiCrust in silico analysis of functional metagenome. (**A**) Main pathways that present changes in the abundance of related genes due to dietary fibre treatment. (**B**) Main pathways that present changes in the non-responder group after dietary fibre treatment. (**A**,**B**) Values of relative abundance above 1.0 indicate an increase in the abundance of related genes after dietary fibre treatment. Paired t-test analysis was performed to estimate significant changes in gene abundance before and after dietary fibre treatment. *P*-values below 0.05 were considered significant. (**C**) Pearson correlation analysis between the changes in feces SCFAs and differential bacterial genus. Correlations with *q*-values below 0.05 after adjustment for multiple analyses are highlighted with an ellipse. The color and slope of the ellipse indicate the magnitude of the correlation, with Pearson’s r value superimposed on the ellipse. The ellipses of negative correlations are shown in red. Correlations with *q*-values > 0.05 are in white.

Finally, a functional analysis was performed to determine if the changes in the bacterial population after dietary fibre treatment may induce variations in bacterial metabolism. Modifications were observed in the relative abundances of genes involved in energy and lipid pathways mainly in the non-responder group (*p*-value of 0.0015 and 0.0022 for energy and lipid metabolism, respectively) (Figure 5A). Specifically, the main pathways that might be affected in the non-responder group were the reduction in glyoxylate and dicarboxylate; penthose-glucuronate; penthose-phosphate; pyruvate; methane; and secondary bile acids metabolism (Figure 5B). Nevertheless, although changes in bacterial and gene abundance were observed, besides *Parabacteroides*, no significant correlation was observed between the differential bacterial population in the responder and non-responder groups and the change in SCFAs in feces after dietary fibre treatment (Figure 5C).

## 4. Discussion

In a previous study from our group, we observed that subjects with hypercholesterolemia compared to subjects with normocholesterolemia were characterized with a higher abundance of *Odoribacter* and a lower abundance of *Anaeroplasma* and *Haemophilus* [6], which were associated with a differential profile in SCFAs. Based on this observation, it was considered necessary to determine the plausibility to modify the microbiota profile and/or functionality of subjects with hypercholesterolemia by dietary fibre supplementation with the end of achieving an improvement in their lipid profile. However, despite the fibre supplementation induced in this study, a favourable change was not observed in the lipid profiles of the majority of volunteers. Only the volunteers who achieved an increase in the content of fecal SCFAs, mainly propionic and butyric acids, were the ones that presented an improvement in their lipid profile. This suggests that microbiota plays an important role in regulating the lipid profile through the generation of SCFAs, as previously suggested; however, not all individuals were capable of increasing the production of SCFAs through an increase in the intake of dietary fibre.

The main hypothesis of the lack of effect could be based on the difference in the response of the microbiota at the individual level from an extra dietary fibre supply. It was observed that two-month dietary fibre supplementation (regardless of fibre type) certainly induced changes in the fecal microbiota profile mainly by an increase in the abundance of *Actinobacteria*, predominantly by the higher abundance of the genus *Bifidobacterium.* Increased abundance of *Bifidobacterium* due to an increase in fibre consumption has been widely reported [9,10,11], and it is considered by many researchers as beneficial in the control of blood lipid profiles. Several studies suggest that *Bifidobacteria* can reduce blood cholesterol levels through its conversion to coprostanol [22]. Similarly, in a randomized placebo-controlled trial, oral administration of *Bifidobacterium longum BB536* for two months was able to induce significant reductions in blood cholesterol levels, which was attributed mainly to the high biliary salt hydrolase activity of *Bifidobacteria* [23], while others have observed that increases in *Bifidobacteriaceae* were associated with amelioration of metabolic syndrome parameters, such as blood lipids, blood pressure, and inflammation [24].

Nonetheless, not all studies have observed the ability of dietary fibre to induce changes in the microbiota profile. For example, the intake of a diet rich in whole-grains (with around 30 g of fibre/day) resulted in higher abundance of *Bifidobacterium* [25], while others indicate that fibre from grains has a minor effect on the intestinal microbiota composition, blood biochemistry, and other health-related parameters [26,27,28]. These differences could be attributed to initial individual differences in the microbiota profile due to external factors. For example, it is suggested that people with habitual low-fibre diets show less ability to change the microbiota profile after dietary fibre supplementation [12,29]. Similar results have been observed in both germ-free animal and in vitro models, where the microbiota from individuals with low-fibre diets show a lower capacity for fibre metabolization [30]. In this respect, in this study, the volunteers who improved their lipid profile had a higher intake of dietary fibre at the beginning of the study (responder group 22.0 g/day, and non-responder group 17.9 g/day). This could suggest that, as mentioned in previous studies, volunteers responding to the treatment could have a colonic microbiota that may present a higher metabolization capacity of additional amounts of fibre.

The increase in the abundance of *Bifidobacterium* was observed in both responder and non-responder groups to dietary fibre treatment. This may suggest that besides the higher abundance of *Bifidobacterium*, other factors or other bacterial genera may be involved in the microbiota capacity to metabolize fibre and to modulate the blood lipid profile. Moreover, dietary fibre supplementation was unable to increase microbiota diversity in both groups (Table 2), which has been observed in a similar way by other human intervention studies with non-digestible carbohydrate diet enrichment [31]. In the present study, microbiota diversity of the responder group remained similar after dietary treatment, while the non-responder group presented a reduction in Shannon and Simpson diversity indexes. This reduction in the diversity of the microbiota was at the expense of SCFA-producing bacteria, such as *Clostridium XIVa* and *Ruminococcus*. Similarly, in addition to the reduction in microbiota diversity, the non-responder group presented an increase in the abundance of genes related to energy metabolism (Figure 5A). This suggests that it is not global gene richness per se, but the abundance distribution of functional genes is also relevant for identifying health-related changes in the gut microbiota.

Some studies suggest that positive responders may have the capacity of structuring a healthier gut ecosystem, possibly through lower gut luminal pH, higher concentrations of butyrate, and stronger competitive exclusion which modify the gut environment to inhibit the detrimental bacteria from carbohydrate fermentation [14]. For example, it has been described that the major group of Gram-negative bacteria found in the human colon, the *Bacteroides* spp., is relatively sensitive to mildly acidic pH [32]. Interestingly, in the present study, it was observed that the non-responder group had a reduction in the ratio of *Firmicutes/Bacteroidetes* (Figure 3B), mainly due to the reduction in the abundance of *Anaerostipes*, *Clostridium XVIa*, *Ruminococcus*, and *Butyricoccus*, which belongs to the *Firmicutes* phylum. This suggests that although the increase in the abundance of *Bifidobacterium* (*Firmicutes*) was induced by dietary fibre, in the non-responder group it was not enough to modulate the gut environment towards a favourable gut ecosystem.

Another important factor to understand how the responder group improved their lipid profile is the finding of the main factor that may predict the outcome. The selection of a bacterial profile that has the highest response in the host is far from easy. The change in *Flavonifractor* was suggested in this study as a plausible predictor of the changes in blood cholesterol, with an accuracy of around 70%. Nevertheless, the understanding of the mechanism of action was not achieved in this study. Initially, it was suggested that the mechanism of action could be related to the increase in SCFAs, mainly propionic and butyric acids. However, no correlation was observed between the changes in *Flavonifractor* and SCFAs, which may suggest that other mechanisms could be involved.

This study presents several limitations. The term “responder” refers primarily to a subject who reacts favourably to therapy, but clearly, there are degrees of responsiveness and different parameters by which the response could be measured. Here, the changes in total blood cholesterol levels were used as the main parameter of responsiveness; nevertheless, other blood lipid profile parameters, such as lipoprotein particle size and its TG and cholesterol content, could also be used as primary response parameters. Host variables’ confounding factors should also be taken into consideration. Recently, using machine-learning strategies, a list of host variables that should be captured in human microbiota studies has been described [33]. Between them, alcohol consumption frequency and bowel movement quality seem to be the main confounding factors that may influence differences in the microbiota profile. Moreover, in this study, it was observed that volunteers classified as responders presented a higher fibre intake at the beginning of the study (Supplementary Table S5), although this difference was not observed after fibre treatment. Additionally, no difference in the amount of the fibre-rich food group’s intake (mainly cereals, legumes, vegetables, and fruits) between responders and non-responders was observed. In this respect, the use of food-frequency questionnaires could help to establish how dietary patterns may influence the observed response in this study. Unfortunately, the focus on these factors was not achieved in this study, and it should be taken into consideration in further studies.

## 5. Conclusions

The main result of this study was the observation that not all volunteers had an improved lipid profile after dietary fibre supplementation. The improvement in the lipid profile was correlated to the changes in SCFAs, mainly propionic and butyric acid, suggesting that the colonic microbiota could influence the response of the extra fibre intake in the change of the lipid profile.

Based on these observations, it was concluded that part of the variability of the response to dietary fibre treatment might be mediated by the response of the microbiota to extra fibre intake, in the sense that volunteers who are responders to fibre treatment tend to maintain more stable diversity and composition of the microbiota able to metabolize dietary fibre, while the volunteers who did not respond to fibre treatment showed a decrease in its diversity. In the latter case, the reduction of SCFAs producing bacterial genera such as *Clostridium XIVa* and *Ruminococcus*, among others, may explain the lack of effectiveness of fibre treatment. However, the mechanisms that lead to the differentiation of bacterial populations in responding and non-responding volunteers due to fibre supplementation are unknown, since according to the observations of this study, both groups started with a similar bacterial population.

## Figures and Tables

**Table 1:** Baseline and two-month treatment values of lipid and SCFA profiles in individuals with a high predominance of *Bacteroides* or *Prevotella*. Data are presented as the mean and 95% confidence interval of the mean (min–max). The paired t-test was used to determine statistical differences of parameters at baseline and 2 months of treatment, while an unpaired t-test was used to determine statistical differences between groups.

Parameter	*Bacteroides* (*n* = 16)			*Prevotella* (*n* = 7)			Difference between Microbiota Profile	
							Baseline	Two-months
	Baseline	Two-months	*p*-value	Baseline	Two-months	*p*-value	*p*-value	*p*-value
Total cholesterol (mg/dL)	212.5 (195.8–229.3)	210.0 (195.2–224.7)	0.6656	209.6 (194.5–224.6)	212.1 (189.6–234.6)	0.7605	0.8072	0.0749
VLDL-Cholesterol (mg/dL)	14.0 (8.8–19.2)	12.8 (8.3–17.3)	0.5313	11.3 (4.9–17.8)	17.0 (6.2–27.8)	0.3040	0.5131	0.3436
IDL-Cholesterol (mg/dL)	11.8 (9.8–13.8)	11.1 (9.0–13.3)	0.2762	10.6 (6.9–14.3)	12.9 (9.5–16.3)	0.6639	0.5068	0.3211
LDL-Cholesterol (mg/dL)	128.3 (115.7–140.9)	127.7 (116.7–138.7)	0.8778	132.9 (123.9–141.9)	131.2 (115.6–146.8)	0.7630	0.6106	0.6916
HDL-Cholesterol (mg/dL)	58.4 (53.0–63.9)	58.3 (53.1–63.6)	0.9530	54.7 (49.0–60.3)	51.0 (44.9–57.0)	0.0012	0.3607	0.0749
Total Triacylglycerides (mg/dL)	116.6 (88.3–144.9)	110.8 (87.1–134.4)	0.5136	96.8 (69.0–124.6)	130.0 (73.8–186.1)	0.2240	0.3487	0.4095
VLDL-Triacylglycerides (mg/dL)	72.1 (48.1–96.0)	68.7 (49.7–87.7)	0.6597	55.9 (33.0–78.7)	84.3 (34.3–134.3)	0.2416	0.3621	0.4288
IDL-Triacylglycerides (mg/dL)	12.3 (10.7–14.0)	11.6 (9.9–13.4)	0.1627	10.9 (8.1–13.6)	12.4 (10.1–14.7)	0.1828	0.2948	0.6018
LDL-Triacylglycerides (mg/dL)	16.3 (13.7–19.0)	15.7 (13.4–18.0)	0.3857	17.1 (14.4–19.8)	17.7 (14.1–21.4)	0.6015	0.6999	0.2804
HDL-Triacylglycerides (mg/dL)	15.8 (13.0–18.6)	14.8 (12.5–17.0)	0.1446	12.9 (10.2–15.6)	15.5 (10.6–20.5)	0.2854	0.1759	0.7149
Particle size								
VLDLz	42.77 (42.45–43.08)	42.56 (42.30–42.81)	0.0712	42.64 (42.17–43.11)	42.65 (42.15–43.15)	0.9707	0.6263	0.6704
LDLz	21.11 (21.03–21.20)	21.08 (21.01–21.15)	0.4079	21.09 (20.94–21.24)	20.96 (20.81–21.11)	0.0070	0.7213	0.0714
HDLz	8.19 (8.18–8.22)	8.20 (8.18–8.22)	0.8589	8.20 (8.16–8.24)	8.18 (8.15–8.21)	0.2073	0.7481	0.2833
Faeces short-chain fatty acids								
Total (mmol/g faeces)	40.7 (27.0–54.4)	46.1 (26.8–65.4)	0.4804	94.5 (56.9–132.1)	106.4 (70.8–142.0)	0.8718	0.0008	0.0015
% Acetic	60.9 (57.4–64.5)	59.5 (56.2–62.8)	0.3210	58.9 (51.2–66.5)	54.9 (50.4–59.4)	0.0908	0.5302	0.0852
% Propionic	20.1 (17.1–23.0)	20.2 (16.9–23.4)	0.9082	19.8 (16.6–23.0)	24.4 (21.7–27.2)	0.0013	0.9123	0.0806
% Butyric	16.7 (13.4–20.0)	18.4 (15.4–21.5)	0.3836	19.0 (13.8–24.1)	18.9 (15.5–22.4)	0.8801	0.4134	0.8342

**Table 2:** Species diversity index in stool samples of volunteers. Data are presented as the mean and 95% confidence interval of the mean (min–max). The paired t-test was used to determine statistical differences of parameters at baseline and 2 months of treatment. *P*-values below 0.05 are highlighted in bold.

Diversity Index	Basal	2 Months	*p*-Value
All volunteers			
OTUs observed	392.2 (373.9–410.5)	384.6 (367.5–401.7)	0.3062
Chao1	442.1 (423.1–461.1)	432.8 (413.8–451.7)	0.2395
Se.Chao1	16.4 (15.1–17.6)	15.9 (14.9–16.8)	0.5761
Shannon	4.01 (3.89–4.12)	3.83 (3.68–3.98)	0.0112
Simpson	0.944 (0.936–0.953)	0.924 (0.907–0.942)	0.0109
Inv.Simpson	23.5 (20.2–26.8)	20.4 (17.4–23.3)	0.1280
Responders			
OTUs observed	391.9 (361.6–422.1)	387.4 (357.7–417.1)	0.6844
Chao1	448.3 (417.2–479.5)	434.0 (400.4–467.6)	0.2748
Se.Chao1	18.4 (16.1–20.6)	15.5 (13.8–17.2)	0.0684
Shannon	3.98 (3.79–4.16)	3.92 (3.67–4.16)	0.5135
Simpson	0.943 (0.929–0.957)	0.930 (0.901–0.960)	0.2363
Inv.Simpson	22.2 (16.7–27.7)	20.9 (16.1–25.76)	0.6644
Non-responders			
OTUs observed	392.5 (368.1–416.8)	382.8 (360.9–404.8)	0.3437
Chao1	438.1 (412.8–463.3)	432.0 (408.8–455.9)	0.5456
Se.Chao1	15.1 (13.6–16.6)	16.1 (15.0–17.2)	0.2752
Shannon	4.03 (3.88–4.18)	3.77 (3.57–3.97)	0.0110
Simpson	0.945 (0.933–0.957)	0.921 (0.898–0.943)	0.0255
Inv.Simpson	24.3 (20.0–28.6)	20.0 (16.0–23.9)	0.1269

## Data Availability

The data presented in this study are available on request from the corresponding author.

## Supplementary Materials

The following are available online at https://www.mdpi.com/article/10.3390/nu14030525/s1. Table S1. Nutritional composition of dietary fibre-rich cookies. Table S2. Demographic characteristics of volunteers included in the study. Table S3. Changes in blood lipid profile (mg/dL) after two months of treatment of wheat bran, psyllium plantago and onion-based fibre. Table S4. Observed changes after 2 months of dietary fibre supplementation in all volunteers. Table S5. Baseline and two months of dietary fibre treatment characteristics between responders and non-responders. Figure S1. Baseline feces microbiota enterotypes.
