# Supplementary material for: Colonic Microbiota Profile Characterization of the Responsiveness to Dietary Fibre Treatment in Hypercholesterolemia
Source: Nutrients. 2022 Jan 25;14(3):525. doi: 10.3390/nu14030525 (PMC8839280; doi:10.3390/nu14030525)

Supplementary Material

Colonic microbiota profile characterization of the responsiveness to dietary fibre treatment in hypercholesterolemia.

Ana Belen Granado-Serrano[1], Meritxell Martín-Garí[1], Virginia Sánchez[2], Marissa Riart Solans[2], Antonia Lafarga Giribets, Rebeca Berdún[1], Ester Vilaprinyó[3], Manuel Portero-Otín M[1] and José CE Serrano[1]*

[1] NUTREN-Nutrigenomics, Department of Experimental Medicine, University of Lleida, Lleida, Spain
[2] Institut Català de la Salut, Catalunya, Spain
[3] Department of Basic Medical Sciences, University of Lleida, Lleida, Spain
* Correspondence: josecarlos.serrano@udl.cat; Tel.: +34-973702408

Table S1. Nutritional composition of dietary fibre-rich cookies. Wheat-bran, Psyllium plantago and Onion-based antioxidant dietary fibre presented the same nutritional composition. Values are presented per 100 g of product. The final weight of each cookie was 15.5 g

Nutrient	Content per 100 g
Energy	329.5 kcal (1386,5 kJ)
Protein	6.2 g
Carbohydrates	51.2 g
Sugars	27.5 g
Starch	23.7 g
Fat	11.1 g
Saturated fat	3.9 g
Monounsaturated fat	5.0 g
Polyunsaturated fat	2.2 g
Dietary fibre	21.0 g

Table S2. Demographic characteristics of volunteers included in the study. Data is presented as mean ± standard deviation, range (min – max) and 95% confidence interval of the mean (min–max). A total of 63 volunteers were included in the study (26 female and 37 male).

Parameter	Mean ± standard deviation	Range (min – max)	95% CI of the mean (min – max)
Age (years)	53.9 ± 7.0	37 - 68	52.1 – 55.7
**Anthropometric parameters**			
Body mass index (kg/m^2)	27.1 ± 3.3	19.2 – 34.6	26.2 – 27.9
Waist circumference			
Female (cm)	89.1 ± 7.4	72.0 – 109.6	86.6 – 91.5
Male (cm)	100.6 ± 7.0	84.5 – 111.0	97.7 – 103.4
Waist/Hip ratio			
Female (cm)	0.87 ± 0.07	0.72 – 1.02	0.85 – 0.89
Male (cm)	0.96 ± 0.04	0.86 – 1.04	0.94 – 0.97
Skinfolds thickness			
Biceps (mm)	11.4 ± 4.9	4.0 – 25.5	10.1 – 12.6
Triceps (mm)	20.5 ± 7.1	6.1 – 37.0	18.7 – 22.3
Subscapular (mm)	21.9 ± 6.5	11.5 – 36.0	20.3 – 23.6
Suprailiac (mm)	16.1 ± 5.6	6.5 – 28.5	14.7 – 17.5
Body fat percentage (%)			
Female (%)	32.5 ± 4.7	24.7 – 42.5	30.9 – 34.1
Male (%)	25.2 ± 4.9	16.4 – 33.3	23.2 – 27.2
**Blood pressure**			
Systolic (mmHg)	129 ± 18	92 - 177	125 – 134
Diastolic (mmHg)	86 ± 10	69 - 112	83 - 88
**Nutritional parameters**			
Energy intake (kcal/day)	1750 ± 412	860 - 3084	1646 – 1853
Protein intake (g/day)	79.6 ± 31.5	25.1 – 208.0	71.6 – 87.5
Carbohydrate intake (g/day)	120.0 ± 61.3	6.8 – 307.0	104.6 – 135.4
Fat intake (g/day)	76.0 ± 28.2	23.4 – 182.0	68.9 – 83.1
Saturated fat (g/day)	23.0 ± 10.8	4.4 – 58.7	20.3 – 25.7
Monounsaturated fat (g/day)	31.8 ± 11.7	7.5 – 60.3	28.8 – 34.8
Polyunsaturated fat (g/day)	10.9 ± 4.8	3.0 – 27.0	9.7 – 12.2
Energy intake distribution			
Protein (%)	17.8 ± 4.6	8.9 – 28.7	16.7 – 18.9
Carbohydrates (%)	41.6 ± 9.4	23.1 – 70.6	39.3 – 44.0
Fat (%)	38.6 ± 8.4	16.2 – 55.9	36.5 – 40.7
Fibre intake (g/day)	19.5 ± 7.0	6.7 – 44.6	17.7 – 21.2
**Blood lipid profile**			
Total cholesterol (mg/dL)	208.5 ± 26.2	158 - 282	201.9 – 215.2
VLDL-Cholesterol (mg/dL)	12.7 ± 8.9	1.9 – 44.8	10.4 – 14.9
IDL-Cholesterol (mg/dL)	10.8 ± 3.7	5.5 – 21.4	9.9 – 11.8
LDL-Cholesterol (mg/dL)	127.0 ± 20.2	90.9 – 191.6	121.9 – 132.1
HDL-Cholesterol (mg/dL)	58.0 ± 1.4	41.2 – 91.5	55.2 – 60.7
Total triacylglycerides (mg/dL)	109.9 ± 48.6	52.6 – 315.9	97.6 – 122.3
VLDL-triacylglycerides (mg/dL)	67.8 ± 43.7	18.5 – 271.6	56.7 – 78.9
IDL-triacylglycerides (mg/dL)	11.6 ± 3.0	6.3 – 18.2	10.8 – 12.3
LDL-triacylglycerides (mg/dL)	15.9 ± 4.3	7.8 – 28.2	14.8 – 17.1
HDL-triacylglycerides (mg/dL)	14.6 ± 4.5	8.0 – 30.6	13.5 – 15.8
Particle size			
VLDL-z	42.74 ± 0.51	41.44 – 43.88	42.61 – 42.87
LDL-z	21.09 ± 0.17	20.38 – 21.48	21.04 – 21.13
HDL-z	8.20 ± 0.50	8.02 – 8.26	8.18 – 8.21
Particle number			
Large VLDL (nmol/L)	1.49 ± 0.74	0.40 – 3.83	1.31 – 1.68
Medium VLDL (nmol/L)	6.94 ± 3.85	1.66 – 23.81	5.96 – 7.91

Small VLDL (nmol/L)	37.7 ± 26.0	10.1 – 156.3	29.1 – 42.3
Large LDL (nmol/L)	115.2 ± 22.6	66.3 – 187.8	109.5 – 121.0
Medium LDL (nmol/L)	341.1 ± 63.0	200.0 – 519.8	325.1 – 357.1
Small LDL (nmol/L)	449.5 ± 77.9	301.2 – 659.9	429.7 469.3
Large HDL (µmol/L)	0.20 ± 0.09	0.40 – 0.70	0.17 – 0.22
Medium HDL (µmol/L)	8.92 ± 2.57	3.16 – 14.7	8.27 – 9.57
Small HDL (µmol/L)	20.03 ± 3.54	13.93 – 31.30	21.13 – 22.93
Other parameters			
NonHDL-p (nmol/L)	918.8 ± 150.9	642 – 1365	880 – 957
Total-p / HDL-p	31.1 ± 6.3	19.4 – 45.3	29.5 – 32.7
LDL-p/HDL-P	29.7 ± 6.1	18.3 – 44.4	28.1 – 31.2
**Faeces Short-chain Fatty acids**			
Total (mmol/g faeces)	52.2 ± 39.7	5.3 – 165.2	41.0 – 63.3
Acetic acid (mmol/g faeces)	32.4 ± 27.4	3.2 – 121.1	24.7 – 40.1
Propionic acid (mmol/g faeces)	10.0 ± 7.7	1.2 – 36.7	7.8 – 12.2
Butyric acid (mmol/g faeces)	9.1 ± 7.7	0.4 – 38.0	6.9 – 11.3
Distribution			
% Acetic acid	60.8 ± 7.9	45.9 – 78.0	58.6 – 63.0
% Propionic acid	19.8 ± 5.2	8.5 – 36.1	18.4 – 21.3
% Butyric acid	16.8 ± 6.2	4.9 – 34.8	15.1 – 18.6

Table S3. Changes in blood lipid profile (mg/dL) after two months of treatment of wheat bran, psyllium plantago and onion-based antioxidant fibre. Data is presented as mean differences (2 months – basal conditions) of each parameter and 95% CI of the mean differences in parenthesis (min to max). The significance of difference observed in each treatment was determined by paired t-test analysis; p-values are included at the right hand of each column. Differences between fibre treatments were determined by one-way anova analysis. Tuckey's multiple comparison tests were performed to determine differences between fibre treatments. P-values below 0.05 were considered as significant differences between the compared variables.

	**Wheat bran fibre**		**Psyllium plantago fibre**		**Onion-based fibre**		
Parameter	*Difference*	*p-value*	*Difference*	*p-value*	*Difference*	*p-value*	*Treatment difference p-value*
Total cholesterol (mg/dL)	4.83 (-6.04 to 15.71)	0.3637	8.90 (-1.74 to 19.54)	0.0964	-0.86 (-9.03 to 7.30)	0.8272	0.3465
VLDL	2.20 (-3.30 to 7.71)	0.4118	-1.04 (-6.14 to 4.06)	0.6746	-1.03 (4.37 to 2.32)	0.5284	0.5130
IDL	1.80 (-0.48 to 4.08)	0.1157	**1.53 (0.51 to 2.57)**	**0.0054**	0.08 (-1.46 to 1.63)	0.4505	0.0794
LDL	2.56 (-4.93 to 10.07)	0.4825	8.40 (-1.35 to 18.15)	0.0875	1.01 (-4.08 to 6.12)	0.6826	0.3300
HDL	-1.74 (-4.72 to 1.24)	0.2371	0.003 (-3.35 to 3.36)	0.9984	-0.32 (-2.89 to 2.26)	0.7995	0.6643
Total triacylglycerides (mg/dL)	13.81 (-14.88 to 42.49)	0.3263	-6.09 (-35.09 to 22.90)	0.6658	-3.98 (-20.28 to 12.32)	0.6161	0.4522
VLDL	10.14 (-13.05 to 33.32)	0.3717	-9.21 (-36.71 to 18.28)	0.4926	-1.05 (-13.90 to 11.80)	0.8664	0.4370
IDL	1.06 (-0.89 to 3.01)	0.2695	**1.17 (0.16 to 2.18)**	**0.0247**	-0.68 (-1.99 to 0.62)	0.2839	0.1525
LDL	1.14 (-1.16 to 3.42)	0.3125	**2.23 (0.49 to 3.96)**	**0.0145**	-0.93 (-2.47 to 0.60)	0.2176	**0.0446**
HDL	1.48 (-1.39 to 4.35)	0.2944	-0.28 (-2.44 to 1.88)	0.7897	-1.30 (-2.97 to 0.37)	0.1197	0.1986
Particle size (nm)							
VLDL-z	-0.12 (-0.32 to 0.07)	0.2052	0.057 (-0.22 to 0.34)	0.6798	-0.179 (-0.389 to 0.039)	0.1022	0.3142
LDL-z	-0.061 (-0.139 to 0.016)	0.1159	0.018 (-0.08 to 0.11)	0.6946	-0.040 (0.091 to 0.011)	0.1174	0.2916
HDL-z	-0.004 (-0.021 to 0.012)	0.5937	-0.003 (-0.027 to 0.020)	0.7470	0.007 (-0.009 to 0.024)	0.3745	0.6209

Table S4. **Observed changes after 2 months of dietary fibre supplementation in all volunteers**. Data are presented as mean and 95% confidence interval of the mean (min – max) (n=63 volunteers). Paired t-test was used to determine statistical differences of parameters at baseline and 2 months of treatment. P-values below 0.05 were considered as a significant difference and highlighted in bold.

Parameter	Baseline	2 months	p-value
**Anthropometric parameters**			
Body mass index (kg/m^2)	27.1 (26.2-27.9)	27.0 (26.2-27.9)	0.4898
Waist circumference	**93.8 (91.5-96.1)**	**95.8 (93.8-98.0)**	**<0.0001**
Waist/Hip ratio	**0.91 (0.89-0.93)**	**0.93 (0.91-0.94)**	**<0.0001**
Skinfolds thickness			
Biceps (mm)	**11.4 (10.1-12.6)**	**12.4 (11.0-13.8)**	**0.0028**
Triceps (mm)	20.5 (18.7-22.3)	21.1 (19.2-22.9)	0.2585
Subscapular (mm)	21.9 (20.3-23.6)	21.3 (19.7-22.9)	0.0821
Suprailiac (mm)	**16.1 (14.7-17.5)**	**17.3 (15.9-18.7)**	**0.0382**
Body fat percentage (%)	**29.5 (28.0-31.0)**	**30.9 (29.4-32.4)**	**0.0034**
**Blood pressure**			
Systolic (mmHg)	129 (125-134)	126 (122-130)	0.0508
Diastolic (mmHg)	**86 (83-88)**	**84 (81-86)**	**0.0106**
**Nutritional parameters**			
Energy intake (kcal/day)	1750 (1646-1853)	1737 (1608-1866)	0.8496
Protein intake (g/day)	79.6 (71.6-87.5)	78.9 (72.3-85.5)	0.8709
Carbohydrate intake (g/day)	120.0 (104.6-135.4)	116.9 (98.9-134.9)	0.5958
Fat intake (g/day)	76.0 (68.9-83.1)	70.3 (63.5-77.1)	0.1821
Saturated fat (g/day)	23.0 (20.3-25.7)	22.4 (19.8-25.1)	0.7189
Monounsaturated fat (g/day)	31.8 (28.8-34.8)	30.4 (27.4-33.2)	0.4502
Polyunsaturated fat (g/day)	10.9 (9.7-12.2)	9.8 (8.8-10.9)	0.1360
Fibre intake (g/day)	19.5 (17.7-21.2)	18.7 (16.8-20.6)	0.4778
Energy intake distribution			
Protein (%)	17.8 (16.7-18.9)	19.0 (17.6-20.4)	0.0828
Carbohydrates (%)	41.6 (39.3-44.0)	41.9 (39.3-44.5)	0.8741
Fat (%)	38.6 (36.5-40.7)	36.7 (34.5-39.0)	0.1722
**Blood lipid profile**			
Total cholesterol (mg/dL)	208.5 (201.9-215.2)	212.8 (206.4-219.2)	0.1249
VLDL-Cholesterol (mg/dL)	12.7 (10.4-14.9)	12.7 (10.4-15.0)	0.9941
IDL-Cholesterol (mg/dL)	10.8 (9.9-11.8)	11.7 (10.7-12.8)	0.0532
LDL-Cholesterol (mg/dL)	127.0 (121.9-132.1)	131.0 (126.2-135.8)	0.0645
HDL-Cholesterol (mg/dL)	58.0 (55.2-60.7)	57.3 (54.7-60.0)	0.4184
Total triacylglycerides (mg/dL)	109.9 (97.6-122.3)	111.0 (99.8-122.2)	0.9264
VLDL-triacylglycerides (mg/dL)	67.8 (56.7-78.9)	67.6 (58.2-76.9)	0.9728
IDL-triacylglycerides (mg/dL)	11.6 (10.8-12.3)	12.1 (11.2-12.9)	0.2233
LDL-triacylglycerides (mg/dL)	15.9 (14.8-17.1)	16.7 (15.6-17.9)	0.1389
HDL-triacylglycerides (mg/dL)	14.6 (13.5-15.8)	14.6 (13.4-15.7)	0.9264
Particle size (nm)			
VLDL-z	42.74 (42.61-42.87)	42.66 (42.53-42.78)	0.2210
LDL-z	21.09 (21.04-21.13)	21.06 (21.01-21.10)	0.2207
HDL-z	8.20 (8.18-8.21)	8.20 (8.18-8.21)	0.9710
Particle number			
Large VLDL (nmol/L)	1.49 (1.31-1.68)	1.49 (1.32-1.68)	0.9928
Medium VLDL (nmol/L)	6.94 (5.96-7.91)	6.80 (5.91-7.69)	0.8121
Small VLDL (nmol/L)	37.7 (29.1-42.3)	35.9 (30.3-41.5)	0.9565
Large LDL (nmol/L)	115.2 (109.5-121.0)	116.2 (110.7-121.7)	0.7412
Medium LDL (nmol/L)	341.1 (325.1-357.1)	353.4 (338.7-368.1)	0.0835
Small LDL (nmol/L)	**449.5 (429.7-469.3)**	**471.5 (451.1-491.9)**	**0.0081**
Large HDL (μmol/L)	0.20 (0.17-0.22)	0.20 (0.18-0.22)	0.8524
Medium HDL (μmol/L)	8.92 (8.27-9.57)	8.81 (8.17-9.45)	0.5707
Small HDL (μmol/L)	20.03 (21.13-22.93)	21.8 (21.0-22.7)	0.6231
Other parameters			

NonHDL-p (nmol/L)	**918.8 (880-957)**	**954.4 (916.8-992.1)**	**0.0233**
Total-p / HDL-p	**31.1 (29.5-32.7)**	**32.56 (30.88-34.24)**	**0.0146**
LDL-p/HDL-P	**29.7 (28.1-31.2)**	**31.10 (29.29-32.72)**	**0.0273**
**Faeces Short-chain Fatty acids**			
Total (mmol/g faeces)	52.2 (41.0-63.3)	50.3 (40.5-60.1)	0.5947
Acetic acid (mmol/g faeces)	32.4 (24.7-40.1)	30.5 (24.2-36.5)	0.5848
Propionic acid (mmol/g faeces)	10.0 (7.8-12.2)	10.2 (8.2-12.3)	0.9430
Butyric acid (mmol/g faeces)	9.1 (6.9-11.3)	9.5 (7.4-11.7)	0.9060
Distribution			
% Acetic acid	60.8 (58.6-63.0)	59.2 (57.2-61.2)	0.6485
% Propionic acid	19.8 (18.4-21.3)	20.6 (19.1-22.1)	0.8335
% Butyric acid	16.8 (15.1-18-6)	17.4 (15.8-19.1)	0.8638

Table S5. **Baseline and two months of dietary fibre treatment characteristics between responders and non-responders**. Data is presented as mean (95% CI of the mean, min–max).). Significant differences within each group was determined by paired t-test analysis; p-values are included at the right hand of each column. Differences between groups (responder vs non-responder) were determined by one-way anova analysis. Tuckey's multiple comparison tests were performed to determine differences between groups. P-values below 0.05 were considered as significant differences between the compared variables.

	Responder	Non-responder	p-value
**Number of volunteers**	24	39	
**Sex (female/male)**	15/9	22/17	0.6335
**Age (years)**	52.3 (49.3-55.2)	54.9 (52.8-57.0)	0.1443

	**Basal conditions**			**2 months of treatment**			**Treatment (p-value)**		**Between Group (p-value)**
Parameter	Responder	Non-responder	p-value	Responder	Non-responder	p-value	Responder	Non-responder	
Anthropometric parameters									
Body mass index (kg/m^2)	27.1 (25.7-28.5)	27.1 (26.0-28.1)	0.9540	27.1 (25.7-28.5)	27.0 (25.9-28.0)	0.9038	0.8704	0.4654	0.7364
Waist circumference	94.9 (91.2-98.6)	93.1 (90.2-96.0)	0.4487	96.4 (92.7-100.0)	95.5 (93.1-98.0)	0.7093	0.0730	**0.0002**	0.3018
Waist/Hip ratio	0.91 (0.88-0.94)	0.90 (0.88-0.93)	0.7438	0.93 (0.90-0.96)	0.92 (0.90-0.94)	0.7416	**0.0172**	**0.0019**	0.9520
Skinfolds thickness									
Biceps (mm)	11.6 (9.5-13.8)	11.2 (9.8-12.6)	0.7489	12.9 (10.5-15.3)	12.1 (10.4-13.7)	0.5507	**0.0108**	0.0642	0.5168
Triceps (mm)	22.2 (19.4-24.9)	19.5 (17.2-21.7)	0.1449	22.0 (19.4-24.7)	20.4 (18.0-22.9)	0.4073	0.8667	0.0750	0.2546
Subscapular (mm)	23.0 (20.0-26.0)	21.3 (19.5-23.1)	0.3137	22.8 (19.8-25.8)	20.4 (18.7-22.0)	0.1282	0.7715	0.0553	0.3074
Suprailiac (mm)	15.7 (13.4-18.0)	16.4 (14.6-18.1)	0.6483	16.9 (14.9-18.9)	17.6 (15.7-19.4)	0.6467	0.1100	0.1466	0.9895
Body fat percentage (%)	31.0 (29.0-33.1)	28.6 (26.6-30.6)	0.1110	31.8 (29.7-34.0)	30.3 (28.3-32.2)	0.3118	0.0509	**0.0163**	0.3205
Blood pressure									
Systolic (mmHg)	127 (120-135)	130 (125-136)	0.5313	128 (121-136)	125 (120-129)	0.4053	0.7608	**0.0029**	**0.0404**
Diastolic (mmHg)	86 (82-90)	86 (83-89)	0.9711	84 (79-88)	84 (80-87)	0.9714	0.0646	0.0656	0.9138
Nutritional parameters									
Energy intake (kcal/day)	1834 (1686-1982)	1698 (1562-1833)	0.2045	1831 (1661-2002)	1679 (1505-1854)	0.2256	0.9743	0.8429	0.9090
Protein intake (g/day)	80.5 (69.8-91-2)	79.0 (68.2-89.8)	0.8499	84.0 (73.6-94.5)	75.7 (67.5-84.0)	0.2254	0.5185	0.5710	0.4224
Carbohydrate intake (g/day)	**151.6 (125.0-178.1)**	**100.5 (84.0-116.1)**	**0.0009**	**146.4 (117.3-175.6)**	**98.8 (78.3-119.2)**	**0.0091**	0.6007	0.8081	0.7733
Fat intake (g/day)	77.3 (67.0-87.6)	75.1 (65.7-84.5)	0.7664	78.2 (67.7-88.6)	65.4 (57.1-73.8)	0.0686	0.9015	0.0845	0.2291
Saturated fat (g/day)	23.7 (18.9-28.5)	22.6 (19.5-25.8)	0.7113	25.2 (20.7-29.7)	20.7 (17.6-23.8)	0.0987	0.5926	0.3039	0.2893
Monounsaturated fat (g/day)	31.2 (27.0-35.3)	32.2 (28.2-36.2)	0.7383	33.4 (28.6-38.1)	28.6 (25.0-32.2)	0.1222	0.4276	0.1343	0.1204
Polyunsaturated fat (g/day)	11.2 (9.3-13.1)	10.8 (9.3-12.3)	0.7511	10.2 (8.8-11.6)	9.6 (8.2-11.1)	0.5777	0.3399	0.2518	0.8894
Fibre intake (g/day)	**22.0 (18.8-25.3)**	**17.9 (16.0-19.7)**	**0.0225**	20.4 (17.5-23.2)	17.7 (15.3-20.1)	0.1748	0.3128	0.8991	0.4893
Energy intake distribution									
Protein (%)	17.4 (15.8-19.0)	18.1 (16.5-19.6)	0.5443	18.3 (16.6-20.1)	19.4 (17.4-21.3)	0.4733	0.3128	0.1640	0.8142
Carbohydrates (%)	42.4 (39.2-45.5)	41.2 (38.0-44.5)	0.6482	41.5 (37.8-45.3)	42.1 (38.7-45.5)	0.8252	0.7066	0.6593	0.5781
Fat (%)	38.1 (35.0-41.3)	38.9 (36.1-41.7)	0.7284	38.2 (34.7-41.7)	35.8 (33.0-38.7)	0.3047	0.9640	0.1018	0.2601
Blood lipid profile									

Total cholesterol (mg/dL)	**222.2 (211.4-232.9)**	**199.9 (193.0-206.8)**	**0.0007**	206.1 (194.8-217.5)	217.0 (210.0-224.0)	0.0975	**<0.0001**	**<0.0001**	**<0.0001**
VLDL-Cholesterol (mg/dL)	13.1 (9.8-16.5)	12.4 (9.4-15.3)	0.7522	10.8 (7.8-13.7)	13.9 (10.8-16.9)	0.1854	0.1037	0.4332	0.1483
IDL-Cholesterol (mg/dL)	**12.4 (10.8-14.0)**	**9.8 (8.8-10.9)**	**0.0077**	11.1 (9.6-12.7)	12.1 (10.8-13.5)	0.3676	**0.0385**	**0.0003**	**0.0001**
LDL-Cholesterol (mg/dL)	**135.9 (128.2-143.7)**	**121.4 (115.5-127.3)**	**0.0047**	126.3 (118.3-134.4)	134.0 (128.4-139.6)	0.1207	**<0.0001**	**<0.0001**	**<0.0001**
HDL-Cholesterol (mg/dL)	60.7 (55.8-65.7)	56.3 (53.3-59.3)	0.1143	57.9 (53.3-62.4)	57.0 (53.9-60.1)	0.7486	**0.0182**	0.5071	**0.0313**
Total triacylglycerides (mg/dL)	113.4 (95.4-131.4)	107.8 (91.7-123.8)	0.6600	99.9 (84.8-115.1)	118.0 (103.2-132.7)	0.1188	**0.0407**	0.3352	0.0978
VLDL-triacylglycerides (mg/dL)	66.9 (51.5-82.4)	68.3 (53.3-83.1)	0.9037	59.0 (46.7-71.3)	73.0 (60.6-85.4)	0.1454	0.1506	0.6181	0.3159
IDL-triacylglycerides (mg/dL)	**12.8 (11.5-14.0)**	**10.8 (10.0-11.7)**	**0.0124**	11.4 (10.1-12.6)	12.5 (11.4-13.7)	0.1965	**0.0079**	**0.0019**	**0.0001**
LDL-triacylglycerides (mg/dL)	**18.1 (16.3-19.9)**	**14.6 (13.4-15.8)**	**0.0018**	15.8 (14.1-17.5)	17.4 (16.0-18.8)	0.1636	**0.0003**	**0.0001**	**<0.0001**
HDL-triacylglycerides (mg/dL)	15.6 (13.5-17.8)	14.0 (12.8-15.2)	0.1636	13.8 (12.3-15.3)	15.1 (13.5-16.6)	0.2898	**0.0070**	0.2536	**0.0246**
Particle size									
VLDL-z	42.82 (42.61-43.03)	42.69 (42.53-42.85)	0.2038	42.72 (42.52-42.93)	42.62 (42.47-42.77)	0.4032	0.2385	0.4556	0.8633
LDL-z	21.13 (21.06-21.19)	21.07 (21.01-21.13)	0.3317	21.08 (21.02-21.14)	21.05 (21.00-21.10)	0.5008	0.1349	0.6088	0.5262
HDL-z	8.21 (8.19-8.22)	8.19 (8.17-8.20)	0.1220	8.21 (8.19-8.22)	8.19 (8.17-8.20)	0.1737	0.7822	0.8923	0.7797
Particle number									
Large VLDL (nmol/L)	1.51 (1.25-1.77)	1.49 (1.23-1.74)	0.9394	1.34 (1.07-1.83)	1.60 (1.37-1.83)	0.1582	0.2127	0.4669	0.1987
Medium VLDL (nmol/L)	6.93 (5.67-8.19)	6.94 (5.58-8.30)	0.9939	7.31 (6.06-8.55)	7.31 (6.06-8.55)	0.1526	0.1157	0.6659	0.2666
Small VLDL (nmol/L)	35.4 (25.7-45.1)	35.9 (27.2-44.6)	0.9425	39.0 (31.5-46.5)	39.0 (31.5-46.5)	0.1630	0.1537	0.5718	0.3049
Large LDL (nmol/L)	**125.3 (116.6-134.0)**	**108.9 (102.2-115.6)**	**0.0044**	112.8 (104.6-120.9)	118.4 (111.3-125.4)	0.3198	**0.0021**	**0.0102**	**0.0001**
Medium LDL (nmol/L)	**370.6 (347.6-393.6)**	**322.4 (303.4-341.4)**	**0.0026**	340.4 (316.7-364.1)	361.6 (343.6-379.5)	0.1636	**<0.0001**	**<0.0001**	**<0.0001**
Small LDL (nmol/L)	**474.0 (441.3-506.8)**	**434.0 (411.1-457.0)**	**0.0481**	449.9 (414.1-485.7)	485.2 (462.2-507.9)	0.0915	**0.0117**	**<0.0001**	**<0.0001**
Large HDL (µmol/L)	0.22 (0.17-0.26)	0.18 (0.16-0.20)	0.1504	0.18 (0.16-0.20)	0.21 (0.18-0.23)	0.1108	0.1378	**0.0202**	**0.0088**
Medium HDL (µmol/L)	9.69 (8.69-10.69)	8.43 (7.63-9.24)	0.0606	9.12 (8.09-10.15)	8.62 (7.82-9.41)	0.4449	0.0647	0.4604	0.0557
Small HDL (µmol/L)	22.47 (20.90-24.04)	21.75 (20.70-22.80)	0.4393	21.10 (19.86-22.34)	22.28 (21.23-23.32)	0.1669	**0.0074**	0.3737	**0.0254**
Other parameters									
NonHDL-p (nmol/L)	**981 (919-1044)**	**879 (836-922)**	**0.0083**	911 (845-977)	982 (940-1024)	0.0658	**<0.0001**	**<0.0001**	**<0.0001**
Total-p / HDL-p	32.2 (29.4-35.0)	30.5 (28.6-32.2)	0.3051	31.7 (28.7-34.6)	33.1 (31.2-35.1)	0.3995	0.3379	**0.0025**	**0.0061**
LDL-p/HDL-P	30.8 (28.1-33.5)	29.0 (27.2-30.8)	0.2610	30.4 (27.6-33.2)	31.6 (29.7-33.5)	0.4740	0.4397	**0.0083**	**0.0188**
Faeces Short-chain Fatty acids									
Total (mmol/g faeces)	54.6 (37.7-71.5)	50.4 (38.3-62.5)	0.7143	57.1 (41.9-72.3)	49.8 (37.3-62.2)	0.5077	0.7612	0.5079	0.5066
Acetic acid (mmol/g faeces)	35.5 (23.4-47.7)	30.2 (22.2-38.3)	0.5019	34.2 (25.0-43.5)	30.2 (22.7-37.7)	0.5496	0.8032	0.5194	0.8019
Propionic acid (mmol/g faeces)	10.0 (7.2-12.9)	9.9 (7.4-12.5)	0.9720	11.2 (8.0-14.4)	10.5 (7.8-13.2)	0.7597	0.4424	0.6858	0.4131
Butyric acid (mmol/g faeces)	8.8 (6.0-11.5)	9.3 (6.7-12.0)	0.8015	10.4 (6.9-13.8)	9.9 (7.2-12.5)	0.8403	0.3024	0.7242	0.3426
Distribution									
% Acetic acid	62.9 (59.6-66.2)	59.3 (57.0-61.7)	0.1139	61.2 (58.0-64.3)	57.7 (55.8-59.7)	0.0887	0.2281	0.2762	0.7782
% Propionic acid	19.8 (17.4-22.1)	19.9 (18.4-21.4)	0.9468	20.2 (18.0-22.5)	20.9 (19.3-22.4)	0.6689	0.5844	0.1212	0.5457
% Butyric acid	15.5 (13.1-17.9)	17.7 (15.7-19.6)	0.2223	16.2 (13.4-19.0)	18.4 (17.0-19.8)	0.1761	0.5812	0.8719	0.7273

Figure S1. Baseline feces microbiota enterotypes. A. and **B.** cluster analysis of microbiota composition at the genus level. Three enterotypes were identified mainly high in *Bacteroides* (cluster 3), high in *Prevotella* (cluster 1) and an intermediate cluster (cluster 2) with mixed composition of *Bacteroides* and *Prevotella*. **C**. and **D**. *Bacteroides* and *Prevotella* abundance in each cluster. **E.** and **F.** Microbiota genus that correlated with *Bacteroides* and *Prevotella* respectively.

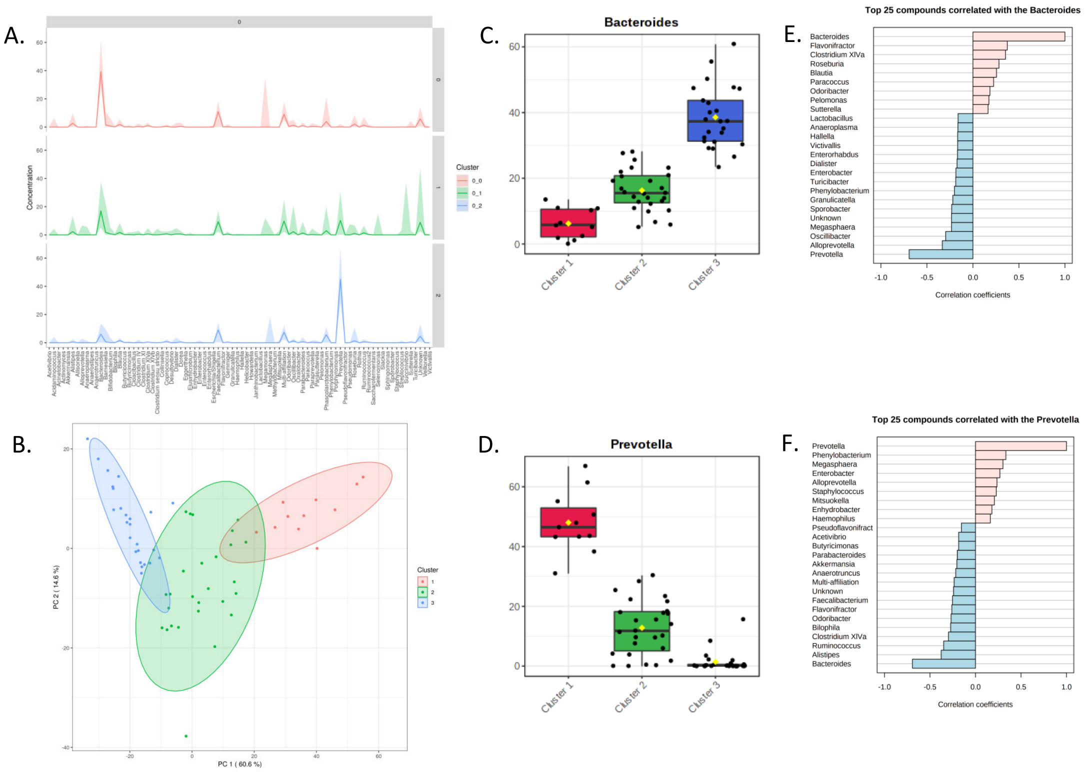

Supplement: Supplementary file 1 [file nutrients-14-00525-s001.zip › nutrients-1543476-supplementary.pdf]
